# JCD Editorial December 2024

**DOI:** 10.1111/jocd.16702

**Published:** 2024-11-28

**Authors:** Michael H. Gold

**Affiliations:** ^1^ Gold Skin Care Center, Tennessee Clinical Research Center Nashville Tennessee USA

It is hard for me to think that 2024 has come and is almost gone. It seems that time is moving faster and faster these days. We all hope that 2024 has been a good year for you and we also only have good things ahead for everyone in 2025.

I would like to spend this page telling everyone how proud we are of this journal. When we embarked on this role as editor, we had big shoes to fill with the leadership that had come before us. What has happened over the past several years has been extraordinary, and with your help, we have seen the impact factor and the prestige of JCD rise higher and higher.

We need to thank all of those people at Wiley, our publisher, who seem to never sleep to ensure that everything we do is timely and up to date. If you review or participate with us, you know they are keen on making sure that we all stay on task—and for the most part, we are always on task. Thank you as well to our amazing Associate Editors—we have the best of the best. I also would be remiss if I did not thank Ms. Angela Brown, my right and left hand, who keeps me on track with everything that is needed to produce this outstanding journal.

In addition, to all of you—all who write and submit papers—we cannot do this without your continued support of this journal. It is because of your trust and dedication that we are where we are today and we will strive to do even more in 2025.

Wishing everyone a wonderful holiday season.

